# Draft Genome Sequence of the Biofilm-Hyperproducing *Acinetobacter baumannii* Clinical Strain MAR002

**DOI:** 10.1128/genomeA.00824-15

**Published:** 2015-07-23

**Authors:** Laura Álvarez-Fraga, María López, María Merino, Soraya Rumbo-Feal, María Tomás, Germán Bou, Margarita Poza

**Affiliations:** Departamento de Microbiología, Instituto de Investigación Biomédica (INIBIC), Complejo Hospitalario Universitario (CHUAC), Universidad de A Coruña (UDC), A Coruña, Spain

## Abstract

We report the draft genome sequence of *Acinetobacter baumannii* strain MAR002, a biofilm-hyperproducing clinical strain isolated during the study CP/09/0033 (GEIH/REIPI-Ab2010, Spain). The genome of *A. baumannii* MAR002 has an approximate length of 3,717,929 bp and 3,300 protein-coding sequences, with a C+G content of 39.09%.

## GENOME ANNOUNCEMENT

*Acinetobacter baumannii* is a nonfermentative Gram-negative coccobacillus. Although this species is a normal inhabitant of the human skin flora, intestinal tract, and respiratory system, it has been shown to cause nosocomial infections, particularly in immunocompromised individuals (1, 2). Biofilm formation is frequent in clinical strains of *A. baumannii* and is an important requirement for chronic colonization of human tissues and persistence in hospital surfaces and medical devices (3, 4). In this study, we report a draft genome sequence of the biofilm-hyperproducing *A. baumannii* strain MAR002, isolated from a wound sample collected from a patient. Genomic DNA was isolated using the Wizard genomic DNA purification kit (Promega) following the manufacturer’s protocols. Genome sequencing was performed using the GS Junior sequencer (454 Life Sequencing Inc., Branford, CT). A whole-genome shotgun fragment library was constructed using the rapid library preparation kit from 500 ng of genomic DNA. The GS Junior Titanium emulsion PCR (emPCR) kit (Lib-L) was used for the amplification of the shotgun library. The GS Junior Titanium sequencing kit combined with the GS Junior Titanium PicoTiterPlate kit was used to determine the nucleotide sequence of the amplified DNA library. Standard 454 pyrosequencing protocols were followed. Reads were assembled into contigs using the 454 gsAssembler software program with default parameters. Contigs were reordered onto the *A. baumannii* ATCC 17978 (NCBI reference sequence no. NC_009085.1), *A. baumannii* AB0057 (NC_011586.1), *A. baumannii* AYE (NC_010410.1) and *A. baumannii* AbH12O-A2 (CP009534.1) reference genomes using the contig ordering tool of the Java-based graphical-interface program Mauve (version 2.3.1) (5, 6). Specific nucleotides were designed for PCR procedures followed by Sanger sequencing in order to close gaps. Genome annotation was performed using the NCBI Prokaryotic Genomes Automatic Annotation Pipeline. PHAST (Phage Search Tool) was used to identify prophage sequences within the *A. baumannii* MAR002 genome (7). A total of 163,265 reads (77,182,857 bp) were generated, with an average length of 541.12 bp, and 99.23% of the reads were assembled. A total of 119 contigs were obtained, 111 of which were large contigs (>500 bp) with lengths between 574 bp and 170,823 bp. The average size of these large contigs was 32,989 bp, and the *N*_50_ was 61,192 bp. After the contig assembly two scaffolds were obtained, scaffold 01 with a length of 2,960,191 bp and a 38.92% G+C content and scaffold 02 with a length of 757,739 bp and a 39.70% G+C content. The estimated complete genome size was 3.72 Mb, with a G+C content of 39.09%. A total of 3,300 protein-coding sequences, 75 pseudogenes, 69 tRNAs, and 6 rRNA clusters were predicted. Using the RAST program, *A. baumannii* AYE, *A. baumannii* ACICU, and *A. baumannii* AB900 were identified as the closest neighbors, with scores of 535, 515, and 492, respectively (8, 9). PHAST analysis revealed a putative intact phage integrated in the genome similar to *Acinetobacter* phage Bphi-B1251 (NC_019541.1), with a length of 54.1 kb, 62 protein-coding sequences, and a G+C content of 36.99%.

### Nucleotide sequence accession numbers.

This whole-genome shotgun project has been deposited at GenBank into two scaffolds under the accession numbers JRHB01000001 and JRHB01000002.
